# Brain and aviation: on the 80th anniversary of Constantin von Economo’s (1876–1931) death

**DOI:** 10.1007/s10072-012-1111-0

**Published:** 2012-05-09

**Authors:** Jarosław Sak, Andrzej Grzybowski

**Affiliations:** 1 Department of Ethics and Human Philosophy, Medical University of Lublin, Szkolna 18, 20-124 Lublin, Poland; 2 Department of Ophthalmology, Poznań City Hospital, ul.Szwajcarska 3, 61-285 Poznan, Poland; 3 Medical Faculty, University of Warmia and Mazury, Olsztyn, Poland

**Keywords:** Constantin von Economo, History of neurology, von Economo neurons, Encephalitis lethargica, Brain

## Abstract

2011 marks the 80th anniversary of the death of Constantin Alexander von Economo who conducted advanced research on the cytoarchitectonics of the brain. This Austrian neurologist and the pioneer of aviation described encephalitis lethargica, discovered the spindle neurons, and postulated the existence of the sleep and wakefulness centre in the brain. What is more he realized two of the biggest dreams of humankind: conquering space and getting to know the secrets of the human brain.

## Introduction

**Fig. 1:**
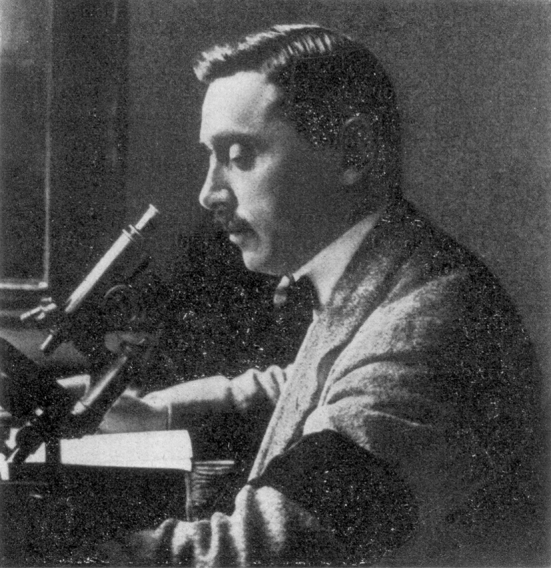
Constantin Alexander von Economo (1876–1931). Reprint from: Spatz H (1931) Constantin von Economo. MMW Munch Med Wochenschr 78:2161–2163

2011 marks the 80th anniversary of the death of an Austrian neurologist and a pioneer of aviation, Constantin Alexander von Economo (1876–1931) (Fig. 1), who independent of Jean René Cruchet (1875–1959) described encephalitis lethargica [1, 2]. He was nominated three times for the Nobel Prize award in Physiology and Medicine [3]. The encephalitis lethargica became the centre element of the book “Awakening” written by an English neurologist, Oliver Sack (it was later made into a movie starring Robert De Niro, and it was awarded three Oscars in 1990). Contemporary neurology and neurophysiology owe much to von Economo’s pioneer research on cytoarchitectonics of the cerebral cortex and the physiology of sleep. He discovered the spindle neurons, which in modern medicine are described as the von Economo neurons (VENs) [4–6]. Constantin von Economo was one of the pioneers of aviation. He was the first Austrian citizen to receive an international pilot license. He even fought as a military pilot during World War I [5]. He had artistic talents and his prints from neurological works are described as little works of art [7, 8].

## Life and work

A detailed biography of von Economo was published together by: a Belgian neurologist Ludo van Bogaert and a French historian of medicine, a nephew of Constantin von Economo, Jean Théodoridès [9]. However, it is worth to mention the most significant events both from his personal and professional life. He was born on 21 August 1876 to an aristocratic family. His father, Johannes Economo (1834–1921) was a Greek industrialist from Edessa who for his deeds became ennobled in 1904. Constantin’s mother Hélène Economo nee Murati (1848–1923) came from Seres [10]. The von Economo family lived on the premises of the Austro-Hungarian Monarchy and adopted the Austrian citizenship a few years before the outbreak of the World War I [11]. As a 14-year-old boy, inspired by the book “The Man of Genius” written by an Italian psychiatrist and criminologist Cesare Lombroso (1835–1909) [12], Constantin von Economo desired to study medicine. In 1901, in Vienna he received his degree of Doctor of Medical Sciences. In 1906, he became the assistant of Julius Wagner-Jauregg (1857–1940) at the Clinic of Psychiatry and Neurology. In 1913, von Economo became an associate professor in psychiatry and neurology and in 1921 he became a full professor. He engaged in treating brain injuries. He studied the cases of Wilson’s disease [13], hemiballismus, multiple sclerosis, paranoia querulans [14] and dipsomania. Economo made an essential observation of a previously unknown disease, encephalitis lethargica, the pandemic of which just broke out in Europe (it lasted between 1917 and 1924).

In 1907, von Economo became interested in aviation and ballooning. In Paris, he learned how to fly a balloon and soon received a balloon pilot license. From 1910, for the next 16 years he was the president of the Österreichischer Aero Club. In 1912, he became the first licensed Austrian aeroplane pilot [9]. During the World War I he served in the air force on the Isonzo Front in the squadron stationed at Lavis. In 1931, shortly before his death, on the occasion of the thirtieth anniversary of the Österreichischer Aero Club, he gave a speech in which he expressed his conviction that in the XX century humankind will overcome gravity and conquer outer space. Merely 2 years earlier (in 1929) Konstantin Eduardovich Tsiolkovsky (1857–1935) developed a theory of multistage rocket motion in the Earth’s gravitational field. It is worth to mention that almost 30 years after von Economo’s speech, in 1961, Yuri Gagarin was set into orbit by a multistage rocket and the flight was successful. On 8 September 1931, after returning from Brno form the First International Neurological Congress, Constantine von Economo suffered from a heart attack complicated by a stroke. On 21 October, von Economo died in his own home in Vienne [15]. He was buried in the family tomb on the St Ann’s Cemetery in Trieste.

## Discovery of the encephalitis lethargica epidemica

In April 1917, von Economo presented a report in front of the Vienna Psychiatric Society in which he described encephalitis lethargica. His article on the subject of this disease was published on 10 May of the same year in the “Wiener Klinische Wochenschrift” [16]. In this article he examined seven cases of this new disease entity. In 1929, he published a monograph devoted to this disease entity [17] and thanks to this: the lethargic encephalitis is also referred to as the “von Economo encephalitis” till this day.

Describing the encephalitis lethargica, Constantin von Economo presented its full symptomatology. He introduced a typology classifying it into three clinical syndromes: somnolent-ophthalmoplegic, hyperkinetic, and amyostatic–akinetic forms. Together with von Wiesner, they succeeded in transferring the disease to primates as a result of incubation of subdural tissue retrieved from a deceased patient [18]. This presented the potentially infectious nature of this disease. They were, however, unable to determine the etiological agent. The time convergence of the pandemic of encephalitis lethargica and the Spanish Flu suggested a common etiological agent of these diseases [19]. Not everybody, however, tended to this hypothesis and von Economo also questioned such an etiology. Especially, since all 13 cases of lethargic encephalitis, which had been previously analyzed by Economo during 1916 and 1917 revealed that none of the patients suffered from any symptoms of the flu. The etiological factor of lethargic encephalitis remained in the sphere of hypotheses until the contemporary times. The introduction of molecular studies to medical diagnostics in 2001 allowed verifying the hypothesis on the common etiological factor of influenza and lethargic encephalitis. The studies using RT-PCR did not indicate the presence of the influenza virus in the archival tissues of patients who suffered from lethargic encephalitis. It was also proven that the Spanish Flu virus could not show neurotropism [20, 21]. The connection between encephalitis and the inflammation of the throat made it possible to propound a hypothesis of a common streptococcal etiological factor [22]. According to the contemporary medical knowledge, lethargic encephalitis may be included in the spectrum of neurological disorders associated with streptococcal infections, along with Sydenham’s chorea and pediatric autoimmune neuropsychiatric disorders associated with streptococcal infections (PANDAS), recently described disorders associated with the streptococcal infections [23].

von Economo as the first scientist described the changes in the substantia nigra of patients deceased due to the chronic encephalitis lethargica. He did this regardless of Konstantin Trietiakow (1893–1956), who presented his doctoral thesis on nigral degeneration as a characteristic feature of Parkinson’s disease in 1919 [24]. Most of the neuropathologists of that time searched for the causes of Parkinson’s disease in the disorder of the cytoarchitectonics of the globus pallidus. Thus, von Economo made a significant contribution to the understanding of the etiology of the disease of the extrapyramidal system [25, 26].

Some articles on the encephalitis lethargica written by von Economo were created as a response to the voices of doubt on his precedence in describing the disease [27, 28]. They were a reaction to the article of Jean-René Cruchet (1875–1959) and his associates, who in 1917, at the same time as Economo, described two cases of encephalitis lethargica [29]. Due to the IWW, the scientific communication between France and Austria was hampered and the cases of this disease were discovered independently in both countries. Cruchet was also unaware of the infectious nature of the disease. What is more, he failed to transfer the experiment on to guinea pigs. In today’s French language writing, encephalitis lethargica is referred to as the “Cruchet–von Economo encephalitis”.

## Impact on contemporary sleep research and discovery of the bipolar spindle neurons

During the studies on encephalitis lethargica, von Economo concluded that there is a centre in the brain that informs about sleep and wakefulness, and he described it as “Schlafsteuerungszentrum” [30]. He was led to this conclusion as a result of observations of patients with encephalitis, with the most common type of this disease (somnolent–ophthalmoplegic), where in addition to the muscle paralysis occurred somnolence of the elevating muscle of the eyelids. He was also aware of the Viennese neuroanatomist and ophthalmologist Ludwig Mauthner (1840–1894), who described the physiological edema of the brain around the aqueduct of Sylvius and the area of the brain associated with sleep. In one of the previous experiences with a cat, in which the lesion of the cerebral peduncle was unintentionally extensive and extended over the interpeduncular fossa, von Economo observed an uninterrupted sleep of the animal for 15 days [31]. On the basis of neuropathological studies, in 1929, von Economo postulated a sleep centre located in the border zone of midbrain and diencephalon [32], rostrally to the oculomotor nucleus, with distinct parts for wakefulness and sleep. His postulates turned out to be correct. After the WWII, experiments revealed that lesion of the hypothalamus induce sleep. Giuseppe Moruzzi (1910–1986) and Horace Magoun (1907–1991) identified the existence of reticular formation, which regulates the awaking/sleeping cycle on the level of the prosencephalon [33].

**Fig. 2:**
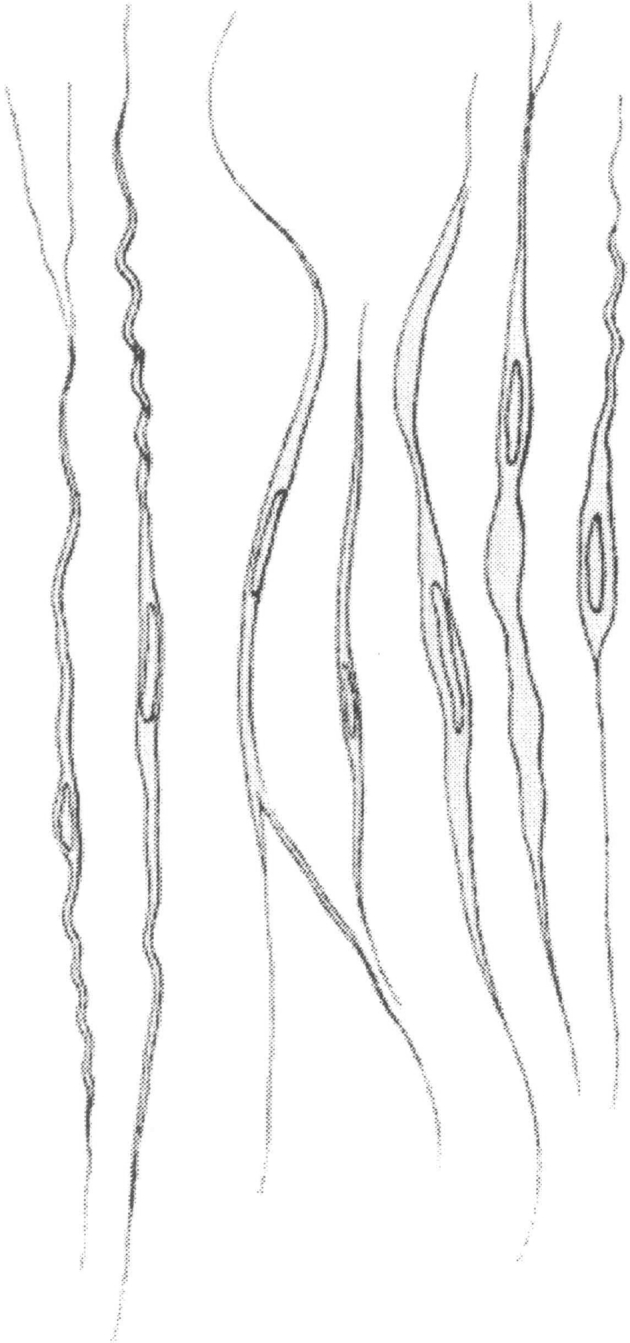
The bipolar spindle neurons (the von Economo neurons). Reprint from: von Economo C (1926) Eine neue Art Spezialzellen des Lobus cinguli und Lobus insulae. Zeitschr Ges Neurol Psychiatr (Berlin) 100:706–712

In 1925, together with George Koskinas (1885–1975), Economo published an atlas of the cytoarchitecture of the human cerebral cortex [34]. The work is valued even today [35]. This magnificent atlas has re-become very precious since the development of in vivo imaging technology that made possible imaging the gross morphology and imaging of brain function. A summary of the major work appeared based on a series of lectures given by Economo in French [36], German [37], Italian [38], and English [39]. A recent re-edition of these lectures has been edited by Triarhou [40] for the English version and by Tomaiuolo, Petrides and Caltagirone for the Italian version [41]. Economo together with Koskinas designed a technique of vertical sections of the brain which allowed presentation of a precise map of the cytoarchitecture of the fields of the cerebral cortex. Economo described large spindle-shaped, bipolar neurons (Fig. 2) located in the frontoinsular and anterior cingulate cortex [42], which are now referred to as the VENs [43, 44]. They are characterized by a spindle-shaped soma, gradually tapering into a single apical axon in one direction, with only a single dendrite facing opposite [45]. These neurons can be found in human brains as well as in the brains of other hominids [46], whales [47, 48] and also in elephants [49]. The existence of other neurons with a similar morphology was also revealed in the dorsolateral prefrontal cortex [50]. The existence of VENs in only the most intelligent mammals became the prerequisite for the hypothesis that they are a sign of neuronal adaptation in very large brains, allowing fast information processing and transfer which is associated with social behaviors. Current scientific reports suggest that the dysfunction of the VENs may be related with the pathogenesis of autism [51, 52], schizophrenia [53] and even suicidal tendencies [54].

## Conclusion

Constantin von Economo, a scientist with versatile interests and extraordinary brightness, co-created the foundation for the modern neurology and neurophysiology. The proofs of the stability of his scientific achievements are the terms with his name contemporarily functioning in the medical literature. He realized two of the biggest dreams of humankind: conquering the space and getting to know the secrets of the brain. von Economo’s activity in brain research as well as in aviation became a prologue to the progress made in subsequent decades. What is more, his prediction concerning the conquest of the outer space was also fulfilled. Modern medical and biological studies added another “chapter” to his “scenario” of research on the brain. The reticular formation became known as being responsible for the awaking/sleeping cycle. Scientists still recognize physiological importance of the spindle neurons which were discovered by von Economo. According to recent reports, these neurons may play an essential role in explaining the neurophysiological mechanisms of genius and madness: the phenomenon that occurred in Lombroso’s book, which inspired von Economo to take up medicine.
